# Association Between Psoriasis and Dementia: Current Evidence

**DOI:** 10.3389/fnagi.2020.570992

**Published:** 2020-10-22

**Authors:** Liu Liu, Si-ting Chen, Hong-jin Li, Yan Qiang, Xiao-ying Sun, Ya-qiong Zhou, Meng Xing, Ying Luo, Yi Ru, Xiao-jie Ding, Le Kuai, Bin Li, Xin Li

**Affiliations:** ^1^Department of Dermatology, Yueyang Hospital of Integrated Traditional Chinese and Western Medicine, Shanghai University of Traditional Chinese Medicine, Shanghai, China; ^2^Shanghai University of Traditional Chinese Medicine, Shanghai, China; ^3^Institute of Dermatology, Shanghai Academy of Traditional Chinese Medicine, Shanghai, China; ^4^Department of Dermatology, Songjiang Hospital Affiliated to Shanghai Jiao Tong University, Jiao Tong University School of Medicine, Shanghai, China

**Keywords:** psoriasis, dementia, systematic review, meta-analysis, psoriatic arthritis

## Abstract

**Background:** Psoriasis and dementia are both inflammatory diseases. The association between psoriasis and dementia has rarely been investigated, and the existing results are conflicting. Thus, we conducted this study to evaluate whether an association exists between psoriasis and dementia.

**Methods:** We searched for studies from six databases from inception to July 30, 2020, using subject and free words. RevMan 5.4 was used to calculate the risk ratio (RR) of dementia in the subjects with psoriasis. When heterogeneity was present, a random-effects model was used. Subgroup, sensitivity, and meta-regression analyses were performed using Stata 15.1.

**Results:** Nine studies were identified and included in the study, of which seven that involved a total of 3,638,487 participants were included in the meta-analysis. We found that among the patients with psoriasis (RR: 1.14, 95% confidence interval [CI]: 1.06–1.24, *p* = 0.0009) and psoriatic arthritis (RR: 2.20, 95% CI: 1.29–3.78, *p* = 0.004), the proportions of those with non-vascular dementia (RR: 1.13, 95% CI: 1.11–1.15, *p* < 0.00001) and vascular dementia (RR: 1.41, 95% CI: 1.09–1.82, *p* = 0.009) were higher than that among the patients without psoriasis. Those with dementia were also more likely to develop psoriasis, and those with severe psoriasis were less likely to die from dementia (RR: 1.88, 95% CI: 0.72–4.90, *p* = 0.020). The meta-regression analysis did not show any significant sources of heterogeneity.

**Conclusions:** The patients with psoriasis and psoriatic arthritis show high prevalence of different types of dementia. Based on the findings of this study, dementia may not be considered a high-risk factor of death from severe psoriasis. However, identification of this potential risk allows for early intervention, thereby reducing comorbidities and deaths.

## Introduction

Psoriasis is a chronic immune-mediated inflammatory skin disease (Menter et al., 2019) that affects 0.5–11.4% of adults and 1.4% of children worldwide (Fu et al., 2018). It is characterized by recurrent erythema and scaly skin accompanied by varying degrees of pruritus (Li et al., 2015). This systemic inflammatory disease negatively affects patients by reducing their quality of life (Innamorati et al., 2016). Dementia, a neurological syndrome defined as a clinical symptom of progressive cognitive impairment, which mainly occurs in the elderly (Lin et al., 2019). Alzheimer's disease (AD) and vascular dementia are the two most common types of dementia. Currently, the pathogenesis of dementia is still unclear. Some studies indicate that its onset is related to aging, family history of the disease, inflammation, genetic susceptibility, immunization, and mild cognitive impairment (Forlenza et al., 2009; Zhang and Jiang, 2015; Yokoyama et al., 2016; Wotton and Goldacre, 2017).

Psoriasis and dementia are both inflammatory and immune diseases (Sardi et al., 2011). In psoriasis, CD4+ T cells differentiate into various types of T cells (including Th1, Th2, Th17, and Treg cells) after inflammatory stimulation (Stadhouders et al., 2018). Further, activated dendritic cells, a kind of antigen-presenting cell, secrete interleukin (IL) 23 and tumor necrosis factor alpha (TNF-α) to induce the activation of Th and Treg cells and the overexpression of inflammatory cytokines such as IL-17, IL-22, and interferon gamma (Jadali and Eslami, 2014; Lowes et al., 2014). These cytokines migrate to the epidermis and act on keratinocytes to participate in the pathogenesis of psoriasis. A study from Germany showed that the peripheral immune system of patients with dementia changed. Whether in Alzheimer's disease, vascular dementia, or frontotemporal dementia, the numbers of B and T cells are reduced (Busse et al., 2017). A recent cohort study compared the relationship between immune system imbalance and the risk of dementia (van der Willik et al., 2019). They used granulocytes and platelets to represent innate immunity and lymphocytes to represent adaptive immunity. The association between innate and adaptive immunities is reflected by three indicators, namely granulocyte-to-lymphocyte ratio (GLR), platelet-to-lymphocyte ratio (PLR), and systemic immune-inflammation index (SII). Results showed that increased risk of dementia was associated with increased granulocyte and platelet counts, whereas decreased risk of dementia was associated with increased lymphocyte count. Increased GLR, PLR, and SII were also associated with increased risk of dementia (including AD and vascular dementia). After the immune system is activated, it secretes inflammatory factors to cause an inflammatory cascade. It is manifested as changes in the expression levels of IL-6, IL-12, IL-23, and other inflammatory cytokines in AD (Heppner et al., 2015) and IL-17A, IL-2, and IL-8 in Lewy body dementia (Surendranathan et al., 2018).

Recently, an increasing number of studies have found a relationship between psoriasis and dementia, with inconsistent results. Therefore, we conducted this research to review and analyze the current evidence, with the aim of investigating the correlation between the two diseases.

## Materials and Methods

### Eligibility Criteria

Articles were included when they met the following criteria: (1) observational research, including cohort, case-control, and cross-sectional studies; (2) any article reporting the association of psoriasis with dementia or dementia-related diseases, with the exact population or incidence/prevalence included; and (3) studies without restrictions according to country and sex.

Articles were excluded when they met any of the following criteria: (1) review articles, experimental studies, randomized controlled trials, or repeated articles; (2) studies without original data; and (3) studies with erroneous/incorrect statistical methods.

### Information Sources and Search Strategy

The PubMed, Embase, China National Knowledge Infrastructure database, Wanfang Data Knowledge Service Platform, the Chinese Scientific Journals Full Text Database (CQVIP), and China Biology Medicine disc (CBMdisc) were searched from their date of inception to July 30, 2020. Subject words and free words, including “psoriasis,” “dementia,” “prevalence,” and “observational study,” were combined during study retrieval. These terms were translated into Chinese and then searched in the Chinese database. We also searched gray literature in the OpenGrey database (www.opengrey.eu).

### Data Extraction and Quality Assessment

The following data were extracted independently by two authors (YQ and XJD): first author, publication year, country of study, study design, diagnostic criteria of psoriasis and dementia, numbers of cases and controls, and mean age. Differences in the extracted data were resolved after discussion with a third author (XL). Two researchers (YL and YR) independently assessed the quality of all the observational studies. For the cohort and case-control studies, the Newcastle-Ottawa Scale (Wells et al., 2000) was applied to evaluate the selection, comparability, and outcome/exposure in each study. The scale score ranged from 0 to 9 points. Studies with scores of ≥ 7 points were considered to have a low risk; 4–6 points, moderate risk; and <3 points, high risk. For the cross-sectional studies, the Agency for Healthcare Research and Quality (AHRQ) tool (Viswanathan et al., 2012; Zeng et al., 2015) was used to assess the risk of bias by answering the 11-item questions with “Yes,” “No,” or “Unclear.” When the proportion of “Yes” answers was high among the items, the risk of bias of the study was considered low. When the two researchers had any disagreement, consensus was reached through discussion.

### Data Analyses

A meta-analysis was performed using RevMan 5.4 and Stata 15.1. Risk ratios (RRs) with 95% confidence intervals (CIs) were calculated for dichotomous data, while mean differences with 95% CIs were summarized for continuous data. *I*^2^ values were used to analyze the extent of heterogeneity. An *I*^2^ value of <50% indicated homogeneity, and a fixed-effects model was applied. An *I*^2^ > 50% indicated substantial heterogeneity; thus, meta-regression, subgroup, and sensitivity analyses were performed to evaluate the possible sources of the heterogeneity. Egger's and Begg's linear regression tests were used to assess publication bias. A *p* < 0.10 in the funnel plot indicated a publication bias.

## Results

### Characteristics of the Pooled Studies

**Figure 1:**
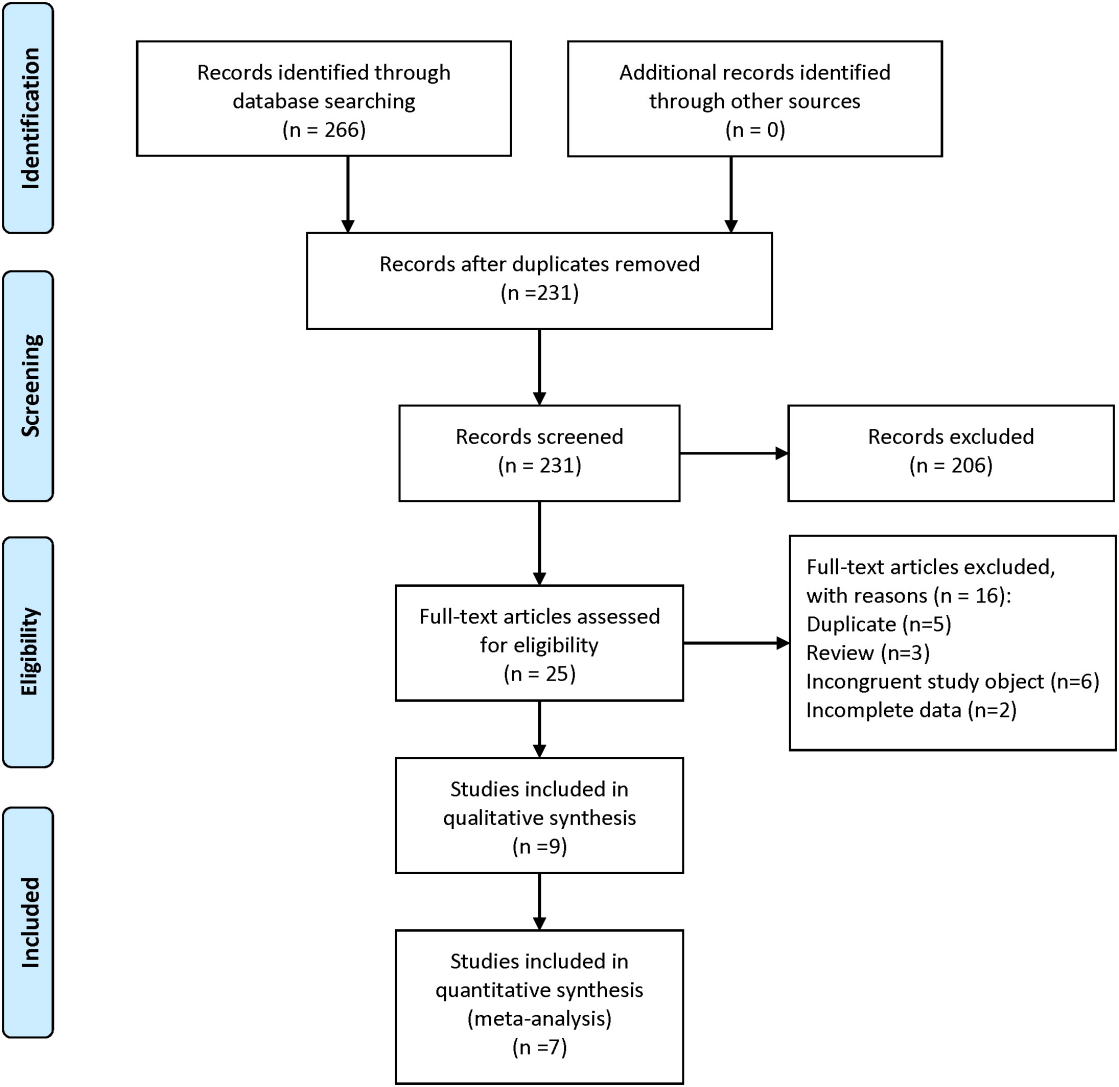
Flowchart of search strategy and study selection according to the 2009 PRISMA guidelines.

A total of 266 articles were retrieved in the initial search. After removing duplicates, 231 articles remained and were screened by their titles and abstracts. Thereafter, 25 studies were thought to be eligible and evaluated for full-text review. Sixteen studies were excluded, and the nine remaining studies (Abuabara et al., 2010; Feldman et al., 2015; Chen et al., 2018; Mitchell et al., 2018; Pezzolo et al., 2018; Huang et al., 2019; Leisner et al., 2019; Lin et al., 2019; Kim et al., 2020) were included. The search strategy and study selection are shown in Figure 1.

Of the nine studies, four were conducted in East Asia (Chen et al., 2018; Huang et al., 2019; Lin et al., 2019; Kim et al., 2020), three in Europe (Abuabara et al., 2010; Pezzolo et al., 2018; Leisner et al., 2019), and two in the USA (Feldman et al., 2015; Mitchell et al., 2018). Five were cohort studies (Abuabara et al., 2010; Pezzolo et al., 2018; Huang et al., 2019; Leisner et al., 2019; Kim et al., 2020); two were case-control studies (Chen et al., 2018; Lin et al., 2019); one was a cross-sectional study (Mitchell et al., 2018), and the remaining one was a retrospective study (Feldman et al., 2015). Almost all the studies used the codes of the *International Statistical Classification of Diseases and Related Health Problems* as a diagnostic basis. The detailed characteristics of these observational studies are shown in Table 1.

**Table 1:** Characteristics of the included studies.

**Studies**	**Study**	**Study**	**Study**	**Diagnostic**	**Total number**		**Age**		**Psoriasis**	**Vascular**		**Dementia-**
	**setting**	**period**	**design**	**criteria**	**(number of dementia cases)**		**(Mean [SD])**		**therapy**	**risk factors**		**related death (Yes/No)**
					**PSO**	**Patients without PSO**	**PSO**	**Patients without PSO**		**PSO**	**Patients without PSO**	
(Abuabara et al., 2010)	U.K. (Centers for Disease Control)	1987–2002	Population-based cohort study	Psoriasis: diagnostic code and a prescription consistent with a severe disease. Dementia: AD (ICD-10 code G30) and Parkinson's disease (ICD-10 codes G20–G21)	321 (7)^a^	862 (10)^a^						Yes
(Feldman et al., 2015)	The United States, the OptumHealth Reporting and Insights claims database	2007–2012	Retrospective study	Psoriasis: (ICD-9-CM code 696.1). Dementia: (ICD-9-CM codes 290, 331.0, 331.1, 331.2).	5,492 (23) Moderate to severe psoriasis	5,492 (12)	47.62 [1.65] y		Biologic therapy: 3,582 (65.2%); Non-biologic systemic therapy: 1,502 (27.3%); Phototherapy, 906 (16.5%)			No
(Chen et al., 2018)	Taiwan, China (LHID2010)	2000–2010	Population-based case-control study	Psoriasis: (ICD-9-CM codes 696); Psoriatic arthropathy: (ICD-9-CM codes 696.0); Dementia: (ICD-9-CM codes 290.0–290.4, 294.1, and 331.0)	19,960 (239); Psoriasis: 221; Psoriatic arthropathy: 18	122,160 (1,174); Psoriasis: 1,124; Psoriatic arthropathy: 50						No
(Pezzolo et al., 2018)	Rotterdam, Netherlands	1990–2010	Population-based prospective cohort study	Psoriasis: a specific algorithm; Dementia: A consensus panel led by a neurologist made the final diagnosis accordingly with standard criteria for dementia and AD.	318 (15) Mild psoriasis: 244 (76.7%); Moderate-to-severe psoriasis: 74 (23.2%)	9,678 (795)	66.86 [8.89] y	66.10 [10.87] y	Topical therapy only: 211 (66.3%); UV therapy: 44 (13.8%); Systemic therapy: 47 (14.7%); No therapy: 60 (18.8%)	Smoking: Current, 87 (28.3%); Past, 137 (44.6%); Never, 83 (27.1%) Body mass index, kgm^−2^: 28.33 ± 4.76 Waist circumference, cm: 96.10 ± 12.26 Total cholesterol level, mmol^−1^:	Smoking: Current, 2,570 (27.0%); Past, 3,966 (41.7%); Never, 2,977 (31.3%) Body mass index, kgm^−2^: 27.64 ± 4.32 Waist circumference, cm: 93.64 ± 12.33 Total cholesterol	No
							5.54 ± 1.02 Systolic blood pressure, mmHg: 144.36 ± 21.63 Diastolic blood pressure, mmHg: 81.14 ± 10.83 Antihypertensive treatment, 150 (47.6%) Hypertension, 176 (58.2%) Diabetes mellitus, 51 (16.0%)	level, mmol^−1^: 5.60 ± 1.02 Systolic blood pressure, mmHg: 142.82 ± 22.19 Diastolic blood pressure, mmHg: 80.89 ± 11.11 Antihypertensive treatment, 3,790 (39.4%) Hypertension, 4,811 (52.4%) Diabetes mellitus, 1,250 (12.9%)				
(Mitchell et al., 2018)	The United States (medical record data repository, >5 million patients)	2001–2017	Cross-sectional study	Psoriasis: (ICD-9 and ICD-10 codes 696.1, L40.0, L40.1, L40.2, L40.3, and L40.4) Dementia: (290, 294, 331, F01, F02, F03, G30, and G31)	5,825 (126)	151,320 (2,965)						No
(Huang et al., 2019)	Taiwan, China (Health Insurance Research Databases, NHIRD, LHID)	1996–2013	Nationwide population-based cohort study	Psoriasis: (ICD-9-CM codes 696.0, 696.1, and 696.8) Dementia: (ICD-9-CM codes 290.0–290.4, 294.1–294.2, 331.0–331.2, and 331.82)	3,820 (245); Vascular dementia: 29; Non-vascular dementia: 216	15,280 (817); Vascular dementia: 91; Non-vascular dementia: 726	40–49: 1,230 (32.2%) 50–59: 1,033 (27%) 60–69: 761 (19.9%) ≥70: 796 (20.8%)	40–49: 4,920 (32.2%) 50–59: 4,132 (27%) 60–69: 3,044 (19.9%) ≥70: 3,184 (20.8%)	Systemic therapy for <90 d: 577 (35 dementia cases); Systemic therapy for ≥90 d: 725 (33 dementia cases); DMARDs and/or biologics: 929 (41 dementia cases); Phototherapy: 373 (27 dementia cases)			No
(Leisner et al., 2019)	Denmark (Danish civil registration system)	1997–2013	Population-based cohort study	Dementia: (ICD-10; ICD-8 codes F00; 290.09, 290.10, F01; 293.09, 293.19, and 290.19)	4,667 (102); Dementia in AD: 63; Vascular dementia: 39	79293						
(1381); Dementia in AD: 939; Vascular dementia: 442						No						
(Lin et al., 2019)	Taiwan, China (Longitudinal Health Insurance Database 2000, LHID2000)	2000–2013	Population-based case-control study	Psoriasis: (ICD-9-CM codes 696/696.1) Dementia: (ICD-9-CM codes 290.0–290.4, 294.1, 331.0–331.2, and 331.82)	7,118 (210)^b^	21,354 (422)^b^	45–49: *n* = 117 (1.6 y) 50–54: *n* = 172 (2.4 y) 55–59: *n* = 281 (4.0 y) 60–64: *n* = 450 (6.3 y) 65–69: *n* = 723 (10.2 y) 70–74: *n* = 1,123 (15.8 y) 75–79: *n* = 1,450 (20.4 y) 80–84: *n* = 1,406 (19.7 y) ≥85: *n* = 1,396 (19.6 y)	45–49: *n* = 351 (1.6 y) 50–54: *n* = 516 (2.4 y) 55–59: *n* = 843 (4.0 y) 60–64: *n* = 1,350 (6.3 y) 65–69: *n* = 2,169 (10.2 y) 70–74: *n* = 3,369 (15.8 y) 75–79: *n* = 4,350 (20.4 y) 80–84: *n* = 4,218 (19.7 y); ≥85: *n* = 4,188 (19.6 y)		Diabetes, 1,940 (27.3%) Hyperlipidemia, 1,776 (25.0%) Hypertension, 4,095 (57.5%) Coronary heart disease, 1,864 (26.2%)	Diabetes, 3,964 (18.6%) Hyperlipidemia, 3,272 (15.3%) Hypertension, 10,593 (49.6%) Coronary heart disease, 4,087 (19.1%)	No
(Kim et al., 2020)	Korea (Korean National Health Insurance System (NHIS) database)	2008–2014	Population-based cohort study	Psoriasis: (ICD-10 codes: L40). Dementia: AD: (ICD-10 codes: F00 or G30)	535,927 (11,311)	2,679,635 (50,209)	No systemic therapy group: 57.71 [11.76]; Systemic therapy group: 55.34 [10.6]	57.55 [11.7] y		a. No systemic therapy group: Diabetes mellitus, 67,741 (13.56%); Hypertension, 160,673 (32.17%); Dyslipidemia, 99,328 (19.89%). b. Systemic therapy group: Diabetes mellitus, 4,742 (13.01%); Hypertension, 10,008 (27.47%); Dyslipidemia, 6,808 (18.68%).	Diabetes mellitus, 281,542 (10.51%); Hypertension, 741,485 (27.67%); Dyslipidemia, 413,393 (15.43%)	

a *Abuabara et al. (2010) reported dementia-related death in patients with severe psoriasis; 321 (7) indicates 7 of 321 patients with severe psoriasis died from dementia, and 862 (10) indicates 10 of 862 control individuals died from dementia*.

b *Lin et al. (2019) reported the number of patients with psoriasis and dementia; 7,118 represents the total number of patients with dementia, and 210 represents that of patients with psoriasis. In the control group, 21,354 patients had no dementia, and 422 had psoriasis*.

*AD, Alzheimer's disease; CON, Control; DMARDs, Disease-modifying antirheumatic drugs; ICD, international Classification of diseases; LHID, Longitudinal Health Insurance Database; PSO, psoriasis; SD: standard deviation; U.K, United Kingdom; UV, Ultraviolet*.

### Risk of Bias

Only Chen et al. (2018) conducted a low-risk research with a score of 7 points. Three studies (Pezzolo et al., 2018; Huang et al., 2019; Lin et al., 2019; Kim et al., 2020) had a modest risk of bias. One study (Leisner et al., 2019) had a high risk with scores of 2 points (Table 2). Only one study had a cross-sectional design. We used the AHRQ tool to assess its risk of bias and found that this study fitted only the first item of the tool (i.e., clarification of the source of data; Table 3) and, therefore, was included/excluded.

**Table 2:** Quality assessment of the cohort and case-control studies using the Newcastle-Ottawa Scale.

**1. Cohort studies**									
**Studies**	**Selection**				**Comparability**	**Outcome**			**Total score**
	**Representativeness of the exposed cohort**	**Selection of the non-exposed cohort**	**Ascertainment of exposure**	**Demonstration that the outcome of interest was not present at the start of study**	**Comparability of cohorts on the basis of the design or analysis**	**Assessment of outcome**	**Follow-up long enough for outcomes to occur**	**Adequacy of follow-up of cohorts**	
(Abuabara et al., 2010)	*	*	*		*				4
(Pezzolo et al., 2018)	*		*		*	*	*	*	6
(Huang et al., 2019)	*	*	*	*	*		*		6
(Leisner et al., 2019)	*		*						2
(Kim et al., 2020)	*		*		*		*		5
**2. Case-control studies**									
**Studies**	**Selection**				**Comparability**	**Exposure**			**Total score**
	**Is the case definition adequate**	**Representativeness of the cases**	**Selection of controls**	**Definition of controls**	**Comparability of cases and controls on the basis of the design or analysis**	**Ascertainment of exposure**	**Same method of ascertainment for cases and controls**	**Non-response rate**	
(Chen et al., 2018)	*	*	*		*	*	*		7
(Lin et al., 2019)	*	*		*	*		*		6

A * means one point and all * of each article are added to get the total score.

**Table 3:** Risk of bias of the cross-sectional studies assessed using the Agency for Healthcare Research and Quality tool.

**Study**	**Term**	**Yes**	**No**	**Unclear**
(Mitchell et al., 2018)	1) Define the source of information (survey, record review)	√		
	2) List inclusion and exclusion criteria for exposed and unexposed subjects (cases and controls) or refer to previous publications		√	
	3) Indicate time period used for identifying patients		√	
	4) Indicate whether or not subjects were consecutive if not population-based			√
	5) Indicate if evaluators of subjective components of study were masked to other aspects of the status of the participants			√
	6) Describe any assessments undertaken for quality assurance purposes (e.g., test/retest of primary outcome measurements)		√	
	7) Explain any patient exclusions from analysis		√	
	8) Describe how confounding was assessed and/or controlled		√	
	9) If applicable, explain how missing data were handled in the analysis		√	
	10) Summarize patient response rates and completeness of data collection		√	
	11) Clarify what follow-up, if any, was expected and the percentage of patients for which incomplete data or follow-up was obtained		√	

### Primary Outcomes

**Figure 2:**
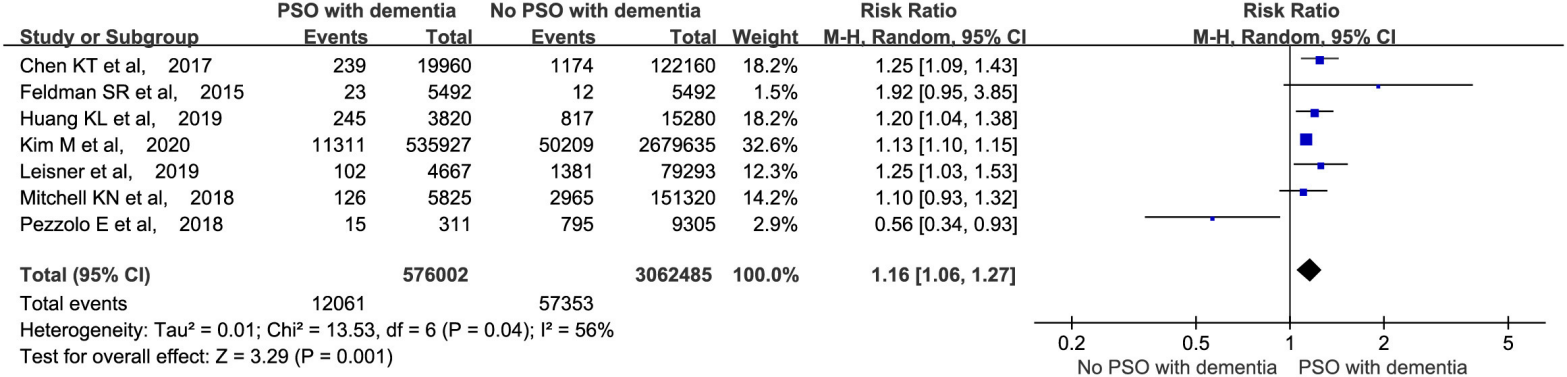
Meta-analysis of the incidence of dementia in patients with psoriasis with the corresponding 95% CI. CI, confidence interval.

Seven studies (Feldman et al., 2015; Chen et al., 2018; Mitchell et al., 2018; Pezzolo et al., 2018; Huang et al., 2019; Leisner et al., 2019; Kim et al., 2020) that involved a total of 3,638,487 participants were included in the meta-analysis. We first analyzed the cases of dementia in all the patients, with and without psoriasis. As shown in Figure 2, a higher proportion of patients with psoriasis had dementia (RR: 1.16, 95% CI: 1.06–1.27, *p* = 0.001; random-effects model) than those without psoriasis.

Meanwhile, Lin et al. investigated the prevalence of psoriasis in patients with and without dementia. Finally, they found that the patients with dementia were at a higher risk of psoriasis (*p* < 0.001), which further confirms the comorbidity of the two disorders from another perspective.

### Meta-Regression Analysis

Owing to the moderate heterogeneity (*I*^2^ = 56%) of the primary outcome, we then performed a meta-regression analysis to examine the possible sources of heterogeneity. The analysis indicated that the variables we predicted were not the possible sources of heterogeneity with no statistical significance (*p* > 0.05; Table 4).

**Table 4:** Meta-regression analysis for evaluating the possible sources of heterogeneity.

**Possible sources of heterogeneity**	**No. of studies**	**Meta-regression coefficient with 95% confidence interval**	***P*-value**
Source of population			0.158
Database	6	1.137 (0.957,1.349)	0.113
Not clear	1	0.563 (0.286, 1.109)	0.081
Considering age			0.087
Yes	5	1.105 (0.915, 1.335)	0.233
No	2	1.224 (0.924, 1.623)	0.123
Differentiated by sex			0.703
Yes	4	1.107 (0.898, 1.365)	0.267
No	3	1.176 (0.838, 1.651)	0.273
Race			0.662
Caucasian	1	1.083 (0.686, 1.709)	0.674
Not clear	6	1.136 (0.860, 1.500)	0.293
Region			0.679
Europe	2	0.961 (0.396, 2.331)	0.907
Asia	3	1.164 (0.544, 2.490)	0.609
America	2	1.219 (0.493, 3.012)	0.576
Study design			0.962
Cohort	4	0.588 (0.083, 4.149)	0.451
Case-control	1	00.121 (0.089, 4.758)	0.541
Cross-sectional	1	0.576 (0.079, 4.316)	0.447
Retrospective study	1	1.538 (0.210, 11.259)	0.541
Study quality			0.743
High quality (≥7 points)	1	1.222 (0.763, 1.958)	0.269
Moderate quality (4–6 points)	3	1.105 (0.834, 1.463)	0.341
Low quality (<4 points)	2	1.145 (0.725, 1.810)	0.414
Severity of psoriasis			0.343
Unclassified	5	1.132 (0.890, 1.440)	0.242
Classified	2	0.849 (0.385, 1.872)	0.617
Psoriatic arthritis included			0.333
Yes	1	2.161 (0.822, 5.677)	0.099
No	7	1.108 (0.926, 1.325)	0.212
Type of dementia			0.536
Vascular dementia	2	1.382 (0.853, 2.241)	0.152
Non-vascular dementia	3	0.106 (0.925, 1.321)	0.216
Uncategorized	4	1.142 (0.886, 1.472)	0.248
Outcome ascertainment			0.558
Examinations	1	0.563 (0.268, 1.182)	0.098
Descriptive data	1	1.083 (0.773, 1.516)	0.549
Chart review	5	1.153 (0.934, 1.424)	0.134

## Secondary Outcomes

### Different Types of Dementia

**Figure 3:**
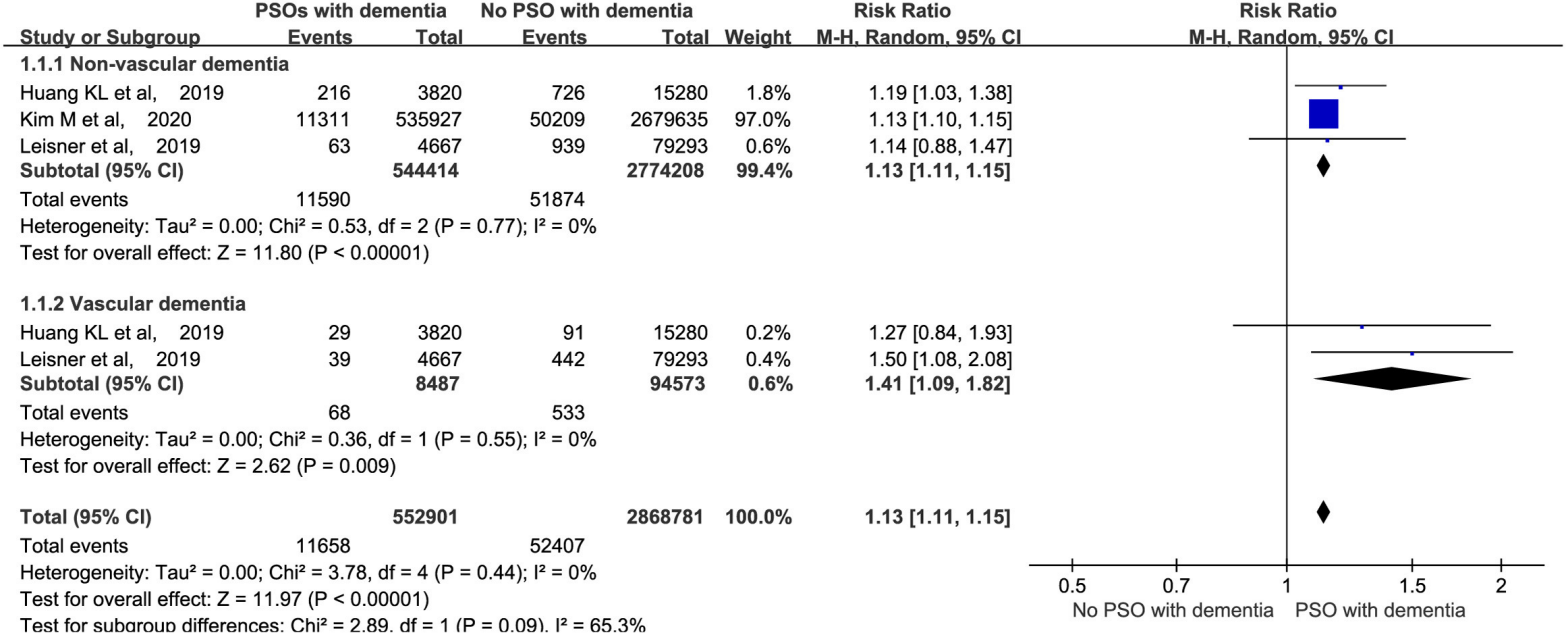
Meta-analysis of the different types of dementia in patients with psoriasis. CI, confidence interval.

A meta-analysis was performed to assess the incidence of non-vascular (including AD) and vascular dementia. The patients with psoriasis had a high probability of developing both types of dementia (*p* < 0.01). The RRs for non-vascular and vascular dementia were 1.13 and 1.41, respectively (Figure 3).

### Comparison of Psoriasis and Psoriatic Arthritis

**Figure 4:**
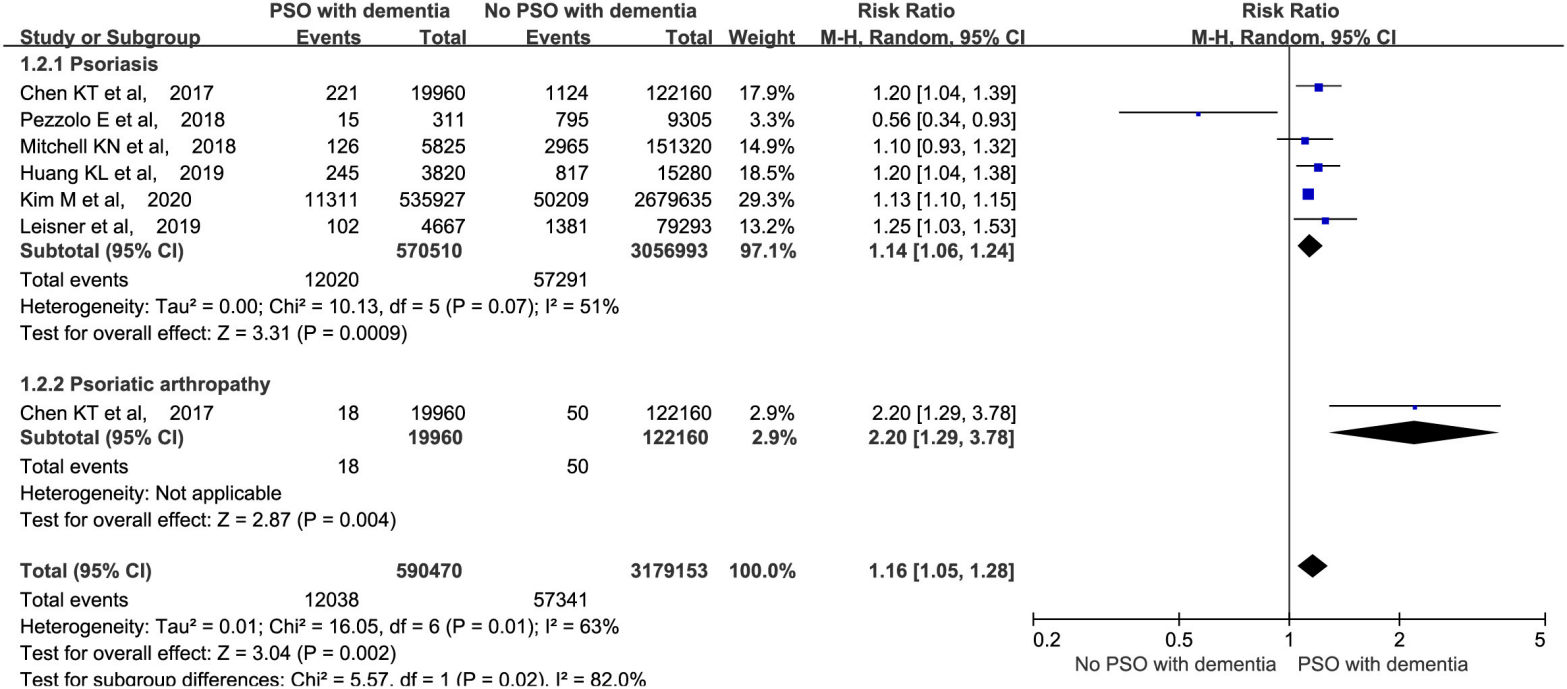
Meta-analysis of dementia in patients with psoriasis and psoriatic arthritis. CI, confidence interval.

We performed a meta-analysis based on whether psoriatic arthritis was included in the studies. The analysis revealed that the patients with psoriasis (RR: 1.14, 95% CI: 1.06–1.24, *p* = 0.0009; random-effects model) and psoriatic arthritis (RR: 2.20, 95% CI: 1.29–3.78, *p* = 0.004; random-effects model) were both at a higher risk of developing dementia (Figure 4).

### Dementia-Related Death

Abuabara et al. (2010) reported no significant difference in the risk of dementia-related deaths among patients with and without psoriasis (*p* = 0.06), which was consistent with our results (*p* = 0.20, data not shown). However, after adjusting for age and sex, the patients with severe psoriasis were 3.64 times more likely to die from dementia than those without severe psoriasis (95% CI: 1.36–9.72).

### Publication Bias

Egger's and Begg's regression tests revealed no publication bias in the pooled studies, with similar *p* values and 95% CIs (*p* = 0.685, 95% CI: −1.578 to 2.211).

### Sensitivity Analysis

In the sensitivity analysis, the study by Pezzolo et al. (2018) might be a source of heterogeneity because when we excluded the study in the meta-analysis, the heterogeneity was reduced (*I*^2^ = 17%). However, the conclusion remained unchanged; that is, the patients with psoriasis had a higher risk of dementia than the patients without psoriasis (RR: 1.16, 95% CI: 1.10–1.21, *p* < 0.00001; Supplementary Figures 1, 2). The reduced heterogeneity can be explained by the fact that only Pezzolo et al. demonstrated that patients with psoriasis are at a lower risk of developing dementia.

## Discussion

To our knowledge, the association between psoriasis and dementia has rarely been investigated, and the existing results are conflicting. Recently, Lam et al. (2020) conducted a systematic review on this topic. They included eight observational studies and found an association between psoriasis and vascular dementia, which was consistent with our findings. Furthermore, we also performed another subgroup analysis, which revealed that the patients with psoriasis and psoriatic arthritis were more susceptible to dementia, regardless of the accompanying type of dementia. The incidence of psoriasis in those with dementia was higher than in those without dementia. Although two studies divided dementia into vascular and non-vascular dementia, they did not report the risk factors related to vascular dementia, including diabetes, hypertension, and metabolic syndrome, which are also risk factors of psoriasis (Li et al., 2016). An interesting result we obtained was that one study reported that dementia-related deaths accounted for most deaths among patients who died from severe psoriasis. However, the meta-analysis revealed no significant difference in the prevalence of severe psoriasis among the patients who died from dementia.

To date, only a few studies have investigated the mechanism of vascular dementia; thus, we can only obtain possible links between AD, one of the most common diseases of non-vascular dementia, and psoriasis from published studies. Moreover, the correlation between them can be explained from three aspects, namely genetics, immunity, and inflammation. A study that analyzed the genetic association of inflammatory diseases with AD revealed a strong relationship between rs2516049, a single-nucleotide polymorphism in the psoriasis gene, and AD, which indicates a genetic overlap between AD and psoriasis, and suggests that the onset of AD is related to the immune process (Yokoyama et al., 2016). Apolipoprotein E (APOE) is the main cholesterol carrier, which is closely related to lipoprotein metabolism, immune regulation, and neural tissue repair (Liu et al., 2013). Several research studies indicated that high expression levels of APOE genes might be an independent risk factor for the occurrence of psoriasis (Al Harthi et al., 2014; Shih et al., 2018). Similarly, the APOE genotypes in AD greatly affect the amyloid beta (Aβ) deposition to form senile plaques and result in cerebral amyloid angiopathy (Liu et al., 2013). APOE4 increases tau phosphorylation and exacerbates the tau pathology in mouse models (Shi and Holtzman, 2018). Therefore, explaining the correlation between the two diseases at a genetic level provides new directions for future research.

Psoriasis and AD are both inflammatory and immune diseases. Colgecen et al. found that patients with psoriasis have impaired visuospatial working memory and executive functions involving the prefrontal cortex because of the underlying ongoing proinflammatory pathology (Colgecen et al., 2016). The IL-23/IL-17 axis plays a significant role in psoriasis. In AD, the IL-12 and IL-23 receptor (p40) expressions on astrocytes and microglia lead to exacerbation of the AD pathology (Vom Berg et al., 2012; Heppner et al., 2015). Mohammadi Shahrokhi et al. (2018) verified that IL-17A expression deteriorates the AD condition through the induction of Aβ and indicated the important roles of the IL-23/IL-17A axis in the AD pathogenesis. Anti-inflammatory drugs used for prolonged periods have been reported to be retained in the body for longer periods, which can retard the progression of AD and delay neurodegeneration (McGeer et al., 2016). TNF-α is a key proinflammatory factor; several pharmacological studies indicated that TNF-α signaling exacerbates both Aβ peptides and tau protein pathologies *in vivo*, which are neuropathological hallmarks of AD (Decourt et al., 2017). Etanercept is a disease-modifying antirheumatic drug (DMARDs) that targets TNF-α. A study showed that etanercept rapidly improved the cognitive function of patients with AD by perispinal administration (Tobinick and Gross, 2008; Decourt et al., 2017). The results of the study by Huang et al. were consistent with the finding that patients with psoriasis who received anti-inflammatory systemic therapy for at least 90 days had a lower risk of dementia, especially those treated with DMARDs and biologics (Huang et al., 2019). Further studies are required to assess the impact of systemic therapy on psoriasis accompanied with dementia.

This study has some limitations. Firstly, only a few articles reported on lifestyle habits and risk factors related to psoriasis and dementia, which affected our comprehensive analysis of the links between the two disorders. Secondly, only one article reported on patients with mild to moderate psoriasis. Therefore, we cannot subdivide the severity of psoriasis and evaluate its relationship with dementia. Thirdly, as only one study reported dementia-related deaths in patients with severe psoriasis, we could not draw a certain conclusion. Therefore, additional similar studies are needed to guide clinical practice in the future. Lastly, the low quality of the involved studies and the fact that only one prospective cohort study was included resulted in weak evidence being included in the meta-analysis and may have biased the results to a certain degree. Further similar analysis-reports including high-level prospective and stratified studies that control confounding factors are required.

## Conclusion

In summary, patients with psoriasis and psoriatic arthritis are at a high risk of developing both non-vascular and vascular dementia. Those with severe psoriasis may not have a higher risk of death from dementia. Clinicians should pay attention to this comorbidity as the identification of this potential risk allows for early intervention to reduce comorbidities and deaths. This study provides a feasible reference for further research on whether the use of preventive medication can reduce the risk of dementia-related death.

## Author Contributions

XL and BL proposed and designed the study. BL obtained funding support. LL, S-tC, MX, and X-yS retrieved and selected the data. YQ and X-jD extracted the data. YL and YR assessed the quality of all the studies. LL, H-jL, LK, and Y-qZ performed all the statistical analyses. LL and S-tC drafted the manuscript and XL revised the manuscript. All authors contributed to the article and approved the submitted version.

## Conflict of Interest

The authors declare that the research was conducted in the absence of any commercial or financial relationships that could be construed as a potential conflict of interest.

## Supplementary Material

Supplementary Figure 1 Sensitivity analysis of the prevalence of dementia in patients with psoriasis, performed using RevMan 5.4. CI, confidence interval.

Supplementary Figure 2 Sensitivity analysis performed using Stata 15.1. CI, confidence interval.

Supplementary File 1 MOOSE Checklist. MOOSE, Meta-analyses Of Observational Studies in Epidemiology.
