# Supplementary figures and images for: Association Between Psoriasis and Dementia: Current Evidence
Source: Front Aging Neurosci. 2020 Oct 22;12:570992. doi: 10.3389/fnagi.2020.570992 (PMC7642958; doi:10.3389/fnagi.2020.570992)

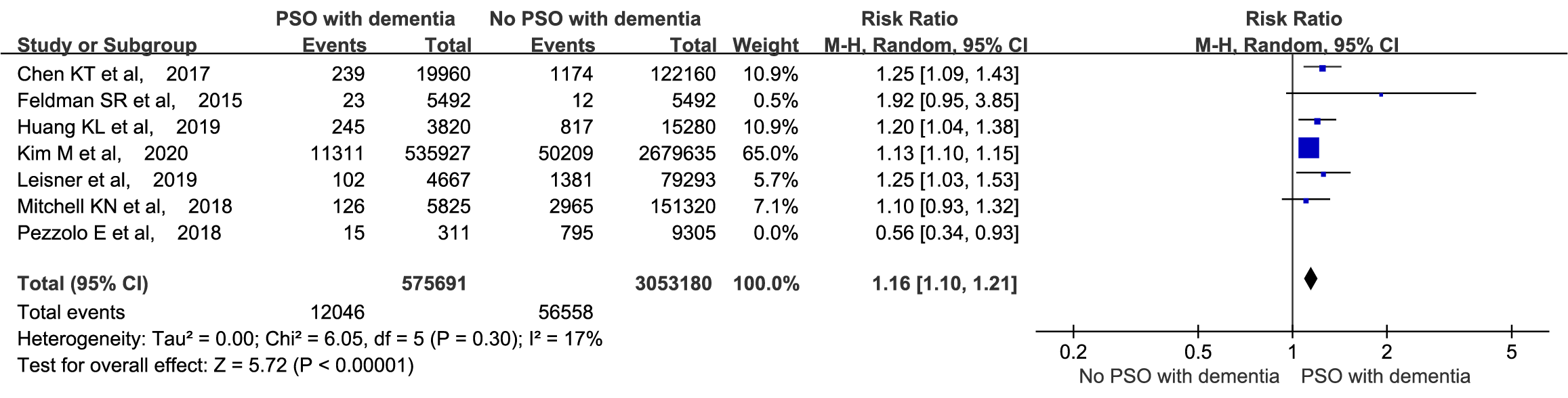

Supplement: Supplementary Figure 1 — Sensitivity analysis of the prevalence of dementia in patients with psoriasis, performed using RevMan 5.4. CI, confidence interval. [file Image_1.TIF]

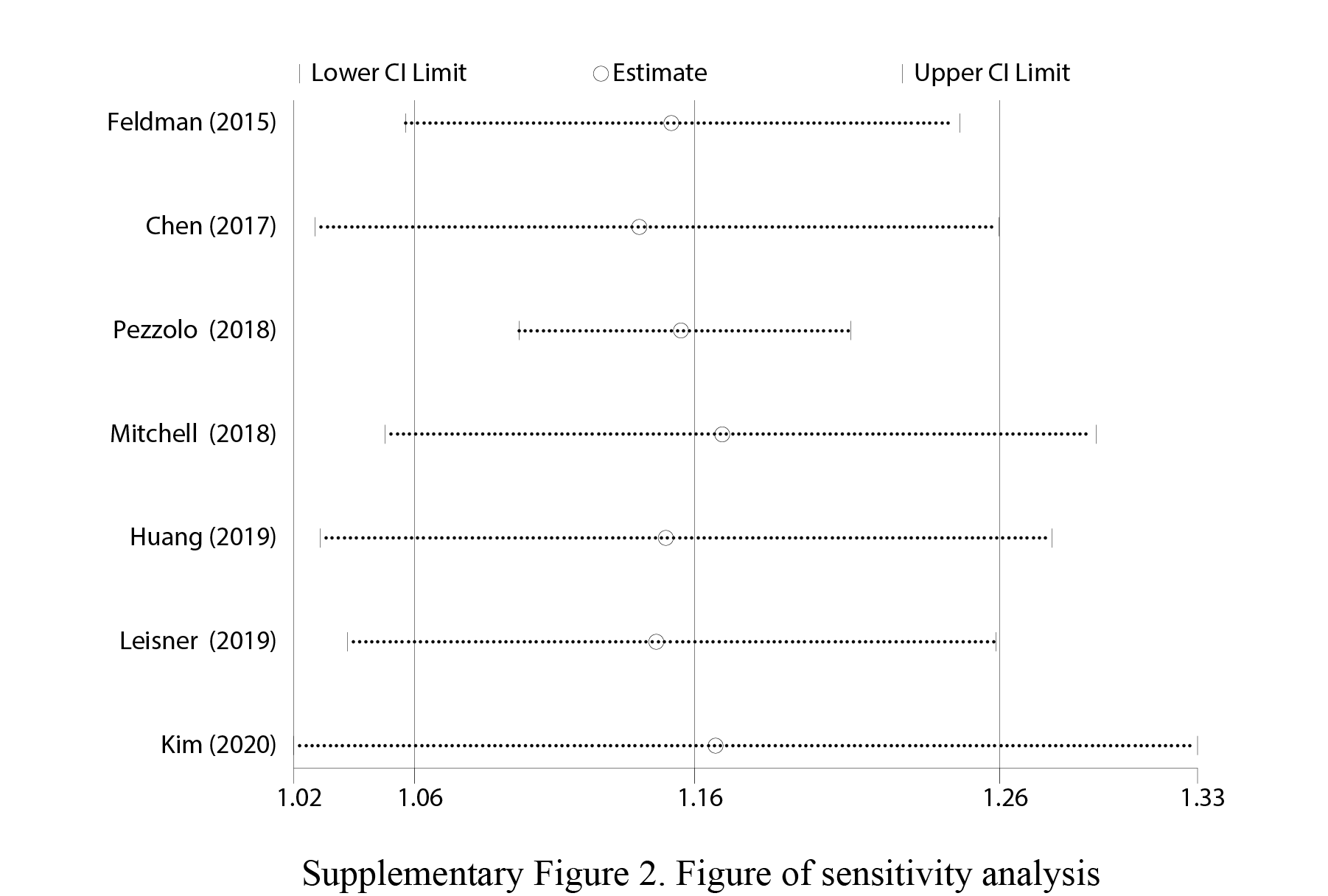

Supplement: Supplementary Figure 2 — Sensitivity analysis performed using Stata 15.1. CI, confidence interval. [file Image_2.TIF]
