# Supplementary material for: Association Between Psoriasis and Dementia: Current Evidence
Source: Front Aging Neurosci. 2020 Oct 22;12:570992. doi: 10.3389/fnagi.2020.570992 (PMC7642958; doi:10.3389/fnagi.2020.570992)

**Supplementary File 1. MOOSE Checklist**

**Association between psoriasis and dementia: current evidence**

Liu Liu^1, 2 ¶^, Si-ting Chen^1, 2 ¶^, Hong-jin Li^3^, Yan Qiang^4^, Xiao-ying Sun^3^, Ya-qiong Zhou^3^, Meng Xing^1, 2^, Ying Luo^1, 2^, Yi Ru^1, 2^, Xiao-jie Ding^1, 2^, Le Kuai^1, 2^, Bin Li^1, 2, 3^, and Xin Li^1, 2, 3^

^1^Department of Dermatology, Yueyang Hospital of Integrated Traditional Chinese and Western Medicine, Shanghai University of Traditional Chinese Medicine, Shanghai 200437, China

^2^Shanghai University of Traditional Chinese Medicine, Shanghai 201203, China

^3^Institute of Dermatology, Shanghai Academy of Traditional Chinese Medicine, Shanghai 201203, China

^4^Department of Dermatology, Songjiang Hospital Affiliated to Shanghai Jiao Tong University, Jiao Tong University School of Medicine, Shanghai, China

^¶^These authors contributed equally.

**Corresponding authors:**

Xin Li

Department of Dermatology, Yueyang Hospital of Integrated Traditional Chinese and Western Medicine, Shanghai University of Traditional Chinese Medicine, Shanghai 200437, China

Phone: +86 13661956326

Fax: +86 021-65162629

Email: 13661956326@163.com

Bin Li

Department of Dermatology, Yueyang Hospital of Integrated Traditional Chinese and Western Medicine, Shanghai University of Traditional Chinese Medicine, Shanghai 200437, China

Phone: +86 18930568129

Fax: +86 021-65162629

Email: 18930568129@163.com

| **Criteria** | | **Brief description of how the criteria were handled in the meta-analysis** |
| --- | --- | --- |
| **Reporting of background should include** | |  |
| √ | Problem definition | Psoriasis and dementia are both inflammatory and immune diseases. Recently, an increasing number of studies have found a relationship between psoriasis and dementia, while the results are inconsistent. |
| √ | Hypothesis statement | Patients with psoriasis are highly associated with dementia |
| √ | Description of study outcomes | Patients with psoriasis have a higher prevalence of dementia.  Additional outcomes: (1) Patients with psoriatic arthritis are at a higher risk of developing dementia, (2) dementia may not be considered a high risk factor of death in cases of severe psoriasis, and (3) patients with dementia are also more likely to develop psoriasis. |
| √ | Type of exposure or intervention used | N/A |
| √ | Type of study designs used | Observational research such as cohort, case-control, and cross-sectional studies were included to assess the prevalence of dementia in the psoriatic population using suitable statistical analyses.  Exclusion: Review articles, experimental studies, or randomized controlled trials. |
| √ | Study population | Patients with psoriasis |
| **Reporting of search strategy should include** | |  |
| √ | Qualifications of searchers | The credentials of the two investigators Ying Luo and Yi Ru are indicated in the author list. |
| √ | Search strategy, including time period included in the synthesis and keywords | Time period: all enrolled databases from their date of construction to July 30, 2020 .  Keywords: psoriasis; dementia; prevalence; observational study . |
| √ | Databases and registries searched | PubMed, Embase, Chinese National Knowledge Infrastructure (CNKI), Wanfang, VIP and China Biology Medicine disc (CBMdisc) . We also searched gray literature in the OpenGrey database (www.opengrey.eu). |
| √ | Search software used, name and version, including special features | We used EndNote X9 to merge retrieved citations and eliminate duplications, and meta-analysis was performed using RevMan 5.4 and Stata 15.1. |
| √ | Use of hand searching | The bibliographies of all retrieved studies were manually searched for further relevant studies. |
| √ | List of citations located and those excluded, including justifications | After screening the full texts of the 25 studies, 16 studies were excluded for the following reasons: 1. five were duplicate studies; 2. three were reviews; 3. six research articles did not match the topic; and 4. two research studies had incomplete data. Details are shown in Fig. 1. |
| √ | Method of addressing articles published in languages other than English | The studies we searched contained English and Chinese articles, but the ultimately articles which enrolled according to the inclusion criteria were English articles. |
| √ | Method of handling abstracts and unpublished studies | We did not contacted authors for abstracts and unpublished studies on the associations of psoriasis with dementia. The studies without original data were excluded. |
| √ | Description of any contact with authors | Contact was not made with authors, as adequate information for the performance of this review was available from studies and abstracts. |
| **Reporting of methods should include** | |  |
| √ | Description of relevance or appropriateness of studies assembled for assessing the hypothesis to be tested | Detailed inclusion and exclusion criteria were described in the study design section. |
| √ | Rationale for the selection and coding of data | Data we analyzed from each of the literatures were associated with the demographic characteristics, study design, exposure and outcome. |
| √ | Assessment of confounding | N/A |
| √ | Assessment of study quality, including blinding of quality assessors; stratification or regression on possible predictors of study results | Two researchers (Y.L. and Y.R.) independently assessed the quality of all the observational studies. For the cohort and case-control studies, the Newcastle-Ottawa Scale was applied in each study. For the cross-sectional studies, the Agency for Healthcare Research and Quality (AHRQ) tool was used to assess the risk of bias. |
| √ | Assessment of heterogeneity | meta-regression, subgroup, and sensitivity analyses were performed to evaluate the possible sources of the heterogeneity. |
| √ | Description of statistical methods in sufficient detail to be replicated | Description of methods of meta-analyses was detailed in the data synthesis and analysis section. |
| √ | Provision of appropriate tables and graphics | We included 1 flow chart, 4 summary tables, 3 figures and 3 supplementary files. |
| **Reporting of results should include** | |  |
| √ | Graph summarizing individual study estimates and overall estimate | Figure 2,3,4 |
| √ | Table giving descriptive information for each study included | Table 1 |
| √ | Results of sensitivity testing | Secondary outcomes analyzed the incidence of psoriasis in different types of dementia、the morbidity of dementia in *Psoriasis and psoriatic arthritis*, and the association with dementia-related deaths in patients with severe psoriasis in this study; effect on heterogeneity is discussed in the literature. |
| √ | Indication of statistical uncertainty of findings | 95% confidence intervals were presented with all summary estimates. |
| **Reporting of discussion should include** | |  |
| √ | Quantitative assessment of bias | Egger’ s and Begg’ s linear regression tests were used to assess publication bias. |
| √ | Justification for exclusion | Papers were excluded on the basis of exclusion criteria listed. We did not systematically exclude any studies on the basis of language or study population size. |
| √ | Assessment of quality of included studies | The Newcastle-Ottawa Scale and Agency for Healthcare Research and Quality *(AHRQ)* tool was used to assess the study quality. Details are showed in Table 2 and 3. |
| **Reporting of conclusions should include** | |  |
| √ | Consideration of alternative explanations for observed results | Only a few articles were confirmed the relationship between lifestyle habits、the severity of psoriasis and dementia, or the association with dementia-related deaths in patients with severe psoriasis. Therefore, to investigate the morbidity and mortality of dementia in severe psoriasis patients, further studies that would include more high-level prospective studies and stratified studies controlling confounding factors are required. |
| √ | Generalization of the conclusions | The patients with psoriasis and psoriatic arthritis show high prevalence of different types of dementia. Based on the findings of this study, dementia may not be considered a high-risk factor of death from severe psoriasis. |
| √ | Guidelines for future research | Investigating the correlation between these two diseases is the first step. Perhaps future research can be conducted on these aspects to unravel the pathogenesis of the two comorbidities, And identification the potential risk of comorbidities allows early intervention, thereby reducing comorbidities and deaths. |
| √ | Disclosure of funding source | Details are provided in the funding source section. |

Funnel plot of log RR for involved studies. The funnel plot shows asymmetry, demonstrating that significant publication bias affects the research in this meta-analysis, and more researches are needed.


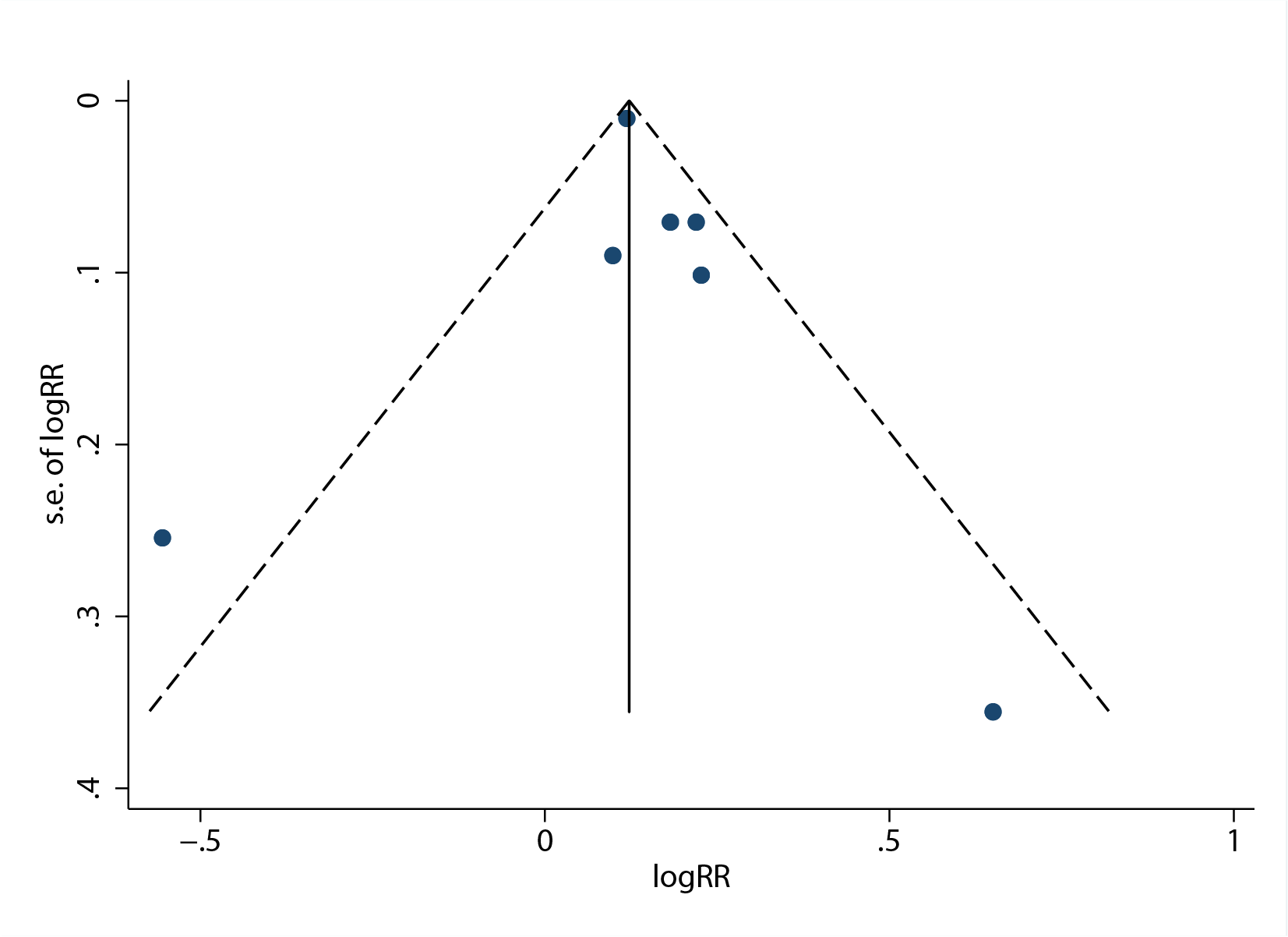

Supplement: Supplementary File 1 — MOOSE Checklist. MOOSE, Meta-analyses Of Observational Studies in Epidemiology. [file Table_1.DOC]
